# Untargeted Metabolomics Analysis of Esophageal Squamous Cell Carcinoma Discovers Dysregulated Metabolic Pathways and Potential Diagnostic Biomarkers

**DOI:** 10.7150/jca.41733

**Published:** 2020-04-06

**Authors:** Zi-Jia Zhu, Zheng Qi, Ji Zhang, Wen-Hua Xue, Li-Feng Li, Zhi-Bo Shen, Ze-Yun Li, Yong-Liang Yuan, Wen-Bin Wang, Jie Zhao

**Affiliations:** 1 Department of Pharmacy, The First Affiliated Hospital of Zhengzhou University, Zhengzhou 450052, China; 2 Henan Key Laboratory of Precision Clinical Pharmacy, Zhengzhou University, Zhengzhou 450052, China; 3 Department of Anesthesiology, The First Affiliated Hospital of Zhengzhou University, Zhengzhou 450052, China; 4 Cancer Center, The First Affiliated Hospital of Zhengzhou University, Zhengzhou, Henan, 450052, China.; 5 Engineering Laboratory for Digital Telemedicine Service, Zhengzhou, Henan, 450052, China.

**Keywords:** metabolomics, esophageal squamous cell carcinoma, diagnostic model, mass spectrometry, plasma biomarkers

## Abstract

**Background**: Esophageal squamous cell carcinoma (ESCC) is one of the most fatal diseases worldwide. Because early diagnosis is difficult, ESCC is mostly diagnosed at an advanced stage, leading to a poor overall prognosis. The purpose of this study was to explore the differences between plasma metabolic profiles in ESCC patients and healthy controls and to establish a diagnostic model of ESCC.

**Methods**: In this study, a cohort of 310 subjects, containing 140 ESCC patients and 170 healthy controls (HC), was recruited. Participants were randomly separated into a training set (80 ESCCs, 80 HCs) and a validation set (60 ESCCs, 90 HCs) and their plasma metabolomics profiles were analyzed by ultra-performance liquid chromatography-tandem quadruple time-of-flight mass spectrometry (UPLC-QTOF/MS) technique. Univariate statistical analysis and multivariate analysis (MVA) methods were used to identify differential metabolites. Finally, the dysregulated pathways associated with ESCC were further explored and the diagnostic performance of the biomarker panel was evaluated.

**Results**: Metabolic analyses identified 34 significant metabolites involved in the metabolism of amino acids, phospholipids, fatty acids, purine, and choline. Farthermore, an effective diagnostic model for ESCC was constructed based on eight metabolites. This panel of biomarkers consisted of hypoxanthine, proline betaine, indoleacrylic acid, inosine, 9-decenoylcarnitine, tetracosahexaenoic acid, LPE (20:4), and LPC (20:5). The model was verified and evaluated in the validation set. The AUC value of the ROC curve was 0.991(95% CI: 0.981-1.000, CI, Confidence interval), with a sensitivity (SE) of 98.8% and a specificity (SP) of 94.9% for the training set and 0.965(95% CI: 0.936-0.993), with a SE of 88.3% and a SP of 88.9% for the validation set. Among them, three biomarkers, indoleacrylic acid, LPC (20:5), and LPE (20:4), exhibited a trend associated with the ESCC progression.

**Conclusions**: Our study identified a novel plasma biomarker panel, which clearly distinguishes ESCC patients and provides insight into the mechanisms of ESCC. This finding may form the basis for the development of a minimally invasive method for ESCC detection.

## Introduction

Esophageal cancer (EC) is one of the most fatal malignancies in the world, causing more than 400000 deaths per year 1. The country most affected by EC is China, with about 90% of the cancer cases being esophageal squamous cell carcinoma (ESCC) 2-4. ESCC is a severe disease that can remain undiagnosed. During the early stage, there are no specific symptoms and by the time of diagnosis, it already reaches an advanced stage. Although the 10-year survival rate of early EC after surgery is higher than 95%, most diagnosed patients are already at advanced stages with a probable 5-year survival rate being only 5%~15% 5. Lugol's chromoendoscopy is the gold standard for EC detection 6. However, its invasiveness and associated discomfort lead to poor patient compliance, which ultimately makes EC detection rate by endoscopy unsatisfactory. Additionally, it requires well trained physicians and expensive equipment, limiting its application in underdeveloped areas 7. Therefore, non-invasive and better adjunctive detection tools with high SE and PE are urgently needed for the diagnosis of ESCC.

Metabolomics focuses on the variation of endogenous small molecule compounds (typically <1000 Da) in biological systems in response to an external stimulus 8-10. Currently, metabolomics has been widely studied for cancer diagnosis, treatment, and prevention 11-14. For instance, a liquid chromatography-mass spectrometry (LC-MS)-based multicenter metabolomics research was carried out to determine more reliable biomarkers for the early diagnosis of hepatocellular carcinoma 15. Mayerle et al. 16 recruited patients for a large-scale study to differentiate pancreatic ductal adenocarcinoma from chronic pancreatitis, achieving high accuracy. In order to elucidate the mechanisms of ESCC onset and discover novel biomarkers, Wang et al. 17 analyzed a relatively large number of early-stage ESCCs by serum LC-MS-based metabolomics. Moreover, Xu et al. 18 discovered a series of potential biomarkers for ESCC diagnostic and therapeutic purposes. In addition, Xu et al. 19 established a LC-MS-based urine metabolomics approach for the early diagnosis of ESCC and the further study of ESCC-related metabolic pathways in the urine. This research uncovered significant perturbations in the metabolic profile of ESCC patients compared with healthy controls (HCs) and identified considerably promising biomarkers. However, the interpretation of metabolic pathway dysregulation in ESCC performed in that study, as well as the SE and SP of the proposed biomarkers require improvement.

In this study, a two-phase biomarker development strategy (training set and validation set) was applied in 310 subjects including clinically relevant controls 20. The plasma levels of metabolites in ESCC patients and HCs were analyzed by ultra-performance liquid chromatography-tandem quadruple time-of-flight mass spectrometry (UPLC-QTOF/MS), a technique that could be used to establish a novel diagnostic tool. After univariate statistical analysis and multivariate data analysis (MVDA), a biomarker panel containing 8 metabolites was identified. The biomarker panel included hypoxanthine, proline betaine, indoleacrylic acid, inosine, 9-decenoylcarnitine, tetracosahexaenoic acid, lysophosphatidylethanolamine (LPE) (20:4), and lysophosphatidylcholine (LPC) (20:5). Finally, the dysregulated metabolic pathways and their biologically relevant functions were studied thoroughly to gain insights into the disturbed metabolism of ESCC.

## Methods

### Chemicals and materials

High performance liquid chromatography (HPLC)-acetonitrile was obtained from Fisher Scientific (Pittsburgh, U.S.A.), and HPLC-grade formic acid was purchased from Fisher Scientific (USA). Ultra-pure water was prepared using the Milli-Q water purification system (Millipore, MA, USA).

### Study design and participants

**Figure 1:**
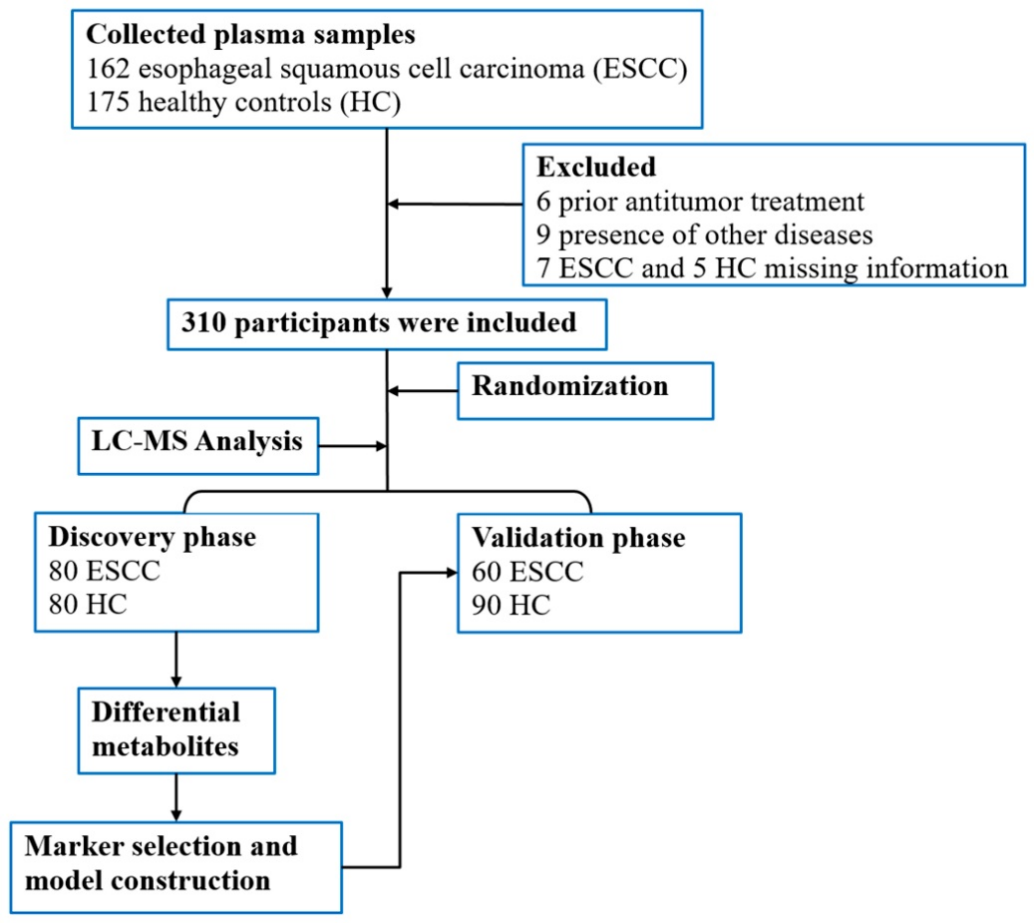
Flow diagram of the study.

A total of 337 participants, including patients with ESCC (n = 162) and HCs (n = 175) above 18 years of age were prospectively selected from the First Affiliated Hospital of Zhengzhou University, Zhengzhou, China for blood sample collection. In total, 310 blood samples were included in the study for metabolomics evaluation. For the ESCC patients, diagnosis was confirmed by pathological examination, and tumor staging was performed based on the American Joint Committee on Cancer (AJCC) 8th staging system 21. Blood sampling and analysis were performed before treatment, which included surgery, chemotherapy, and radiotherapy. Additionally, participants with missing clinical information or other diseases such as hypertension, diabetes, and metabolic syndrome were excluded. The study design and flow diagram is shown in Figure 1.

### Plasma sample collection and pretreatment

Blood samples were collected from the recruited subjects before 8 am and after fasting, stored in K2-EDTA vacutainer tubes, and cooled down at 4°C immediately. The samples were centrifuged at 4000 rpm for 10 min at 4°C within 2 hours. The supernatants (plasma) were separated and transferred into new 2-mL cryotubes, and immediately stored at -80°C until analysis. Directly before analysis, the samples were thawed on ice. Then, 200 μL of pre-cooled acetonitrile was added to 50 μL of plasma, the mixture was vortexed for 30 s, and centrifuged at 14,000 rpm for 10 min at 4°C. After that, 200 μL from the supernatant was transferred to a clean tube, dried under a vacuum concentrator at room temperature, dissolved with 100 μL water/acetonitrile (1:1) solution, vortexed for 30 s and centrifuged at 14000 rpm for 10 min at 4 °C. The supernatant was separated and subjected to metabolite analysis by UPLC-QTOF/MS. Quality control (QC) samples were prepared with equal amounts of plasma (10 μL) from 100 randomly selected plasma samples. QC measurements were performed periodically, after every 7 samples, to ensure the stability of the large-scale analysis.

### LC-MS analysis

The acquisition was performed using an ExionLC™ AD system coupled to a X500R QTOF system (all devices from AB SCIEX, Framingham, U.S.A.). A Waters ACQUITY HSS T3 column (100 × 2.1 mm, 1.8 μm) was used in positive electrospray ionization mode (ESI^+^). The reconstituted sample (3 μL) was injected for chromatography separation at 30 °C, with a flow rate of 0.4 mL/min, 0.1% formic acid in HPLC water as mobile phase A and HPLC acetonitrile as mobile phase B. A 14-min linear gradient was set as follows: 0 min: 5% B, 0-1 min: 5-30% B, 1-3 min: 30-60% B, 3-10 min: 60-95% B, 10-12 min: 95% B, 12-12.1 min: 95-5% B, 12.1-14 min: 5% B.

The SCIEX X500R QTOF system with a Turbo V™ source and capable of electrospray ionization (ESI) was used in positive polarity. The ion source temperature was set to 600ºC and the ion source voltage was set to 5500 V. An information dependent acquisition (IDA) method, consisting of a TOF-MS survey (50-1000 Da for 150 msec) and up to 10 dependent MS/MS scans (50-1000 Da for 35 msec), was carried out to acquire high resolution mass data. The declustering potential (DP) was set to 80V. The collision energy (CE) was set to 40 eV with a collision energy spread (CES) of ± 20 eV. To achieve the most complete MS/MS coverage, the dynamic background subtraction (DBS) function was activated.

### Data processing

The raw data were aligned, deconvoluted, and normalized (sum of total area) using the MarkerView™ Software 1.3 (SCIEX). The retention time (RT) was from 0.5 min to 14 min. The mass and RT tolerance values were set to 10 ppm and 0.15 minutes, respectively. After the 80% rule was used to treat the missing values for each sample group, a list of the intensities for each detected peak was generated, using retention time and the mass-to-charge (m/z) ratio data pairs as the parameters for each ion. Thus, each spectral feature was represented by a unique m/z, retention time, and peak area. After the data preprocessing, the resulting 2-dimensional data matrix (pareto scaled) was subjected to MVA using the SIMCA-P software (version 14.1, Umetrics AB, Umea, Sweden). Principal component analysis (PCA) was used to visualize system stability of the system and sample distribution. The orthogonal partial least squares discriminant analysis (OPLS-DA) was used to identify the variables responsible for the discrimination. The "goodness of fit" and predictive power of the model were evaluated using R2Y (sum) and Q2 (sum), respectively. The variable importance in the projection (VIP) was used to identify significant features. A 200-times permutation test was performed to evaluate the risk of model overfitting.

Additionally, the Student's t-test was applied to measure the significance of each variable. To remove any *p*-values (up to a 95 % confidence) that could have been false positives, the resultant *p* values for each metabolite were corrected by Bonferroni correction. Volcano plot, S-plot, and Venn diagram depictions were used to filter important variables that displayed statistical significance (adjusted *p* < 0.05), significant fold changes (FC > 1.2 or < -1.2), and VIP > 1 between the two groups. The Formula Finder algorithm was used to identify potential differential metabolites and generate a group of probable formulas on an unknown ion based on the secondary fragment information, mass error, and isotope distribution patterns. Subsequently, the HMDB, METLIN, MoNA, and KEGG databases were browsed for these candidates, and the final decisions about possible structures and final biomarkers were based on the obtained MS/MS spectra. Statistical analyses were performed using the SPSS software version 21.0 (IBM Corp., Armonk, New York). By stepwise regression analysis, the factors with significant influence were selected as independent variables, and an “optimal” regression equation was established to find potential biomarkers for distinguishing between the ESCC group and HC groups. Logistic regression analysis and receiver operating characteristic (ROC) analysis were used for the diagnosis of ESCC and HC. The area under the receiver-operating characteristic curves (AUROC) was calculated by SPSS to evaluate the predictive performance of the constructed signatures in both the training and validation sets. The Youden index (*J*) was used as the best threshold to select the optimal cut-point that maximized its value 22. A heat map of the identified key metabolites was drawn by the pheatmap package (R version 3.3.0). Open database sources, including the KEGG and MetaboAnalyst, were used to identify metabolic pathways. By this analysis, several dysregulated metabolic pathways associated with the development of ESCC were uncovered.

## Results

### Clinical characteristics of patients

After diagnosis according to strict pathological criteria and a well-defined exclusion process, a total of 140 ESCC and 170 HC samples were included and randomly divided into a training set (80 ESCC, 80 HC) and a validation set (60 ESCC, 90 HC). The detailed characteristics of the study participants are summarized in Table 1, including age, gender, cancer staging category, routine blood work, and biochemical information. As expected, the incidence of ESCC was higher in men and positively correlated with age. There was no statistical difference in white blood cell (WBC) counts for both the training (*p* = 0.9632) and the validation (*p* = 0.391) sets. However, the level of hemoglobin (Hb) was significantly lower in ESCC patients than in HCs (*p* < 0.01). Similar to Hb, platelet count (PLT) was also significantly decreased in ESCC patients (*p* < 0.01).

### Screening and Defining of Differential Metabolites

**Figure 2:**
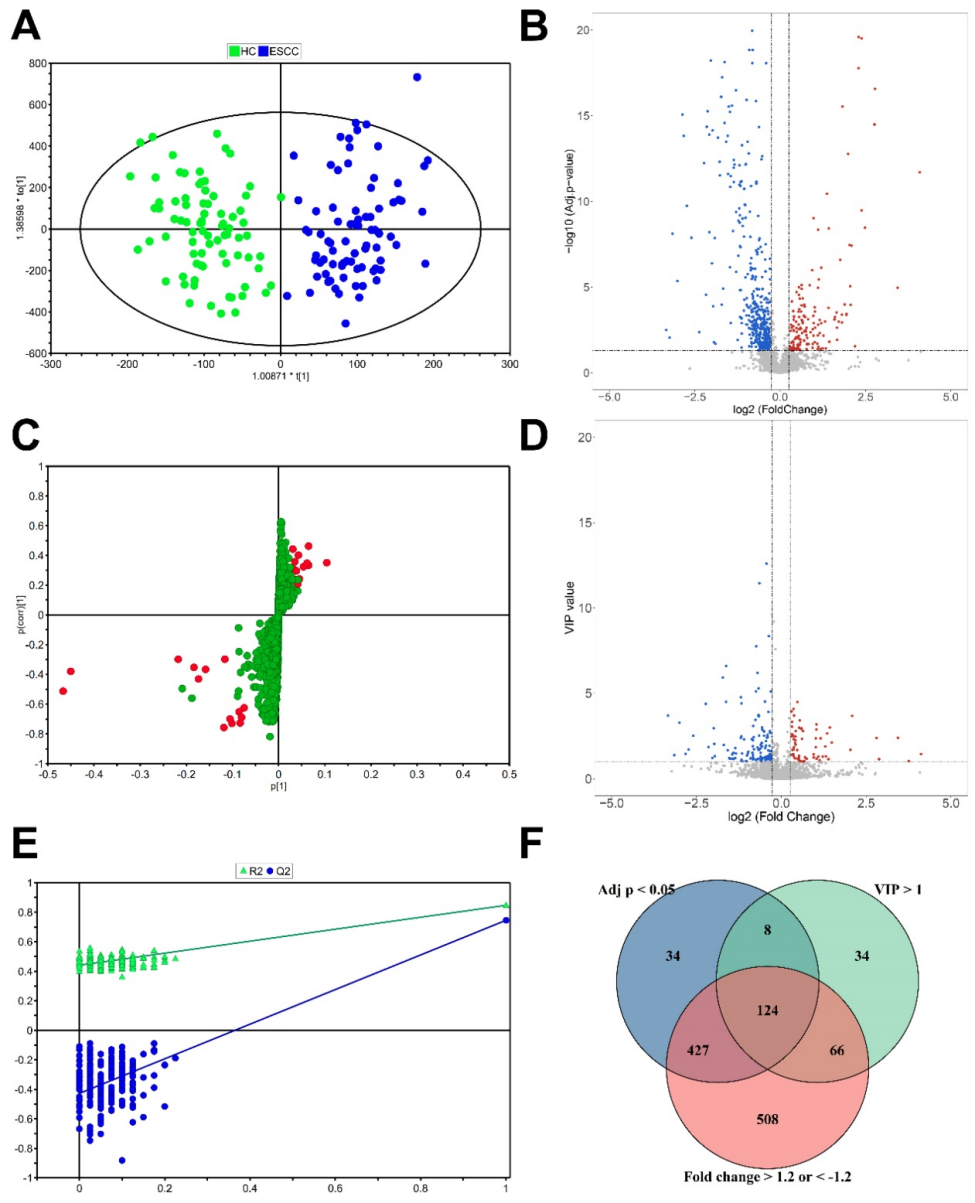
** Statistical analysis for the diagnosis of ESCC.** (A) OPLS-DA score plot of the training set. (B) Volcano plot of VIP values. (C) S-plot of the OPLS-DA model for the HC and ESCC. (D) Volcano plot of adjusted p values. (E) validation plot obtained from 200 permutation tests. (F) Venn diagram of adjusted *p* value, VIP, and fold change results.

A total of 3339 ions were detected in the training set by UPLC-Q/TOF-MS operating at the positive ion mode. The QC samples formed a tight cluster in the score plots (Figure S1A, B), with a width no larger than twice the standard deviation (SD), confirming the stability and reliability of the experimental approach. The OPLS-DA score plots were used to estimate the holistic distribution of plasma samples from both the training and validation sets (Figure 2A, S1C). The OPLS-DA score plot of the training set showed a clear separation. The evaluation parameters of the obtained OPLS-DA models are: R2X = 0.429, R2Y = 0.809, and Q2Y = 0.725, indicating that the models have high predictive power. These well differentiated patterns verify the robustness of this approach. S-plot showed the typical metabolites in OPLS models (Figure 2C). Response permutation testing (RPT) is a method for evaluating the accuracy of OPLS-DA models. After 200 times of modeling and replacement verification (Figure 2E), the regression equation of the obtained model and the resulting R2Y and Q2Y values, (0.0, 0.431) and (0.0, -0.411), respectively, show that the model is stable and reliable, without overfitting. Three filters, including VIP >1.0, adjusted* p* < 0.05, and FC > 1.2 or < -1.2, were applied to determine differential metabolites. Accordingly, a total of 124 features were selected based on results of rudimentary filtering of volcano plots (Figure 2B, D) and the Venn diagram (Figure 2E). From these 124 differential variables, 34 metabolites were identified and used for subsequent pathway analysis. Eleven metabolite standards were purchased to verify potential differential metabolites. The detail information of these metabolites is shown in Table S1. Take the identification of inosine as an example (Figure S2).

**Figure 3:**
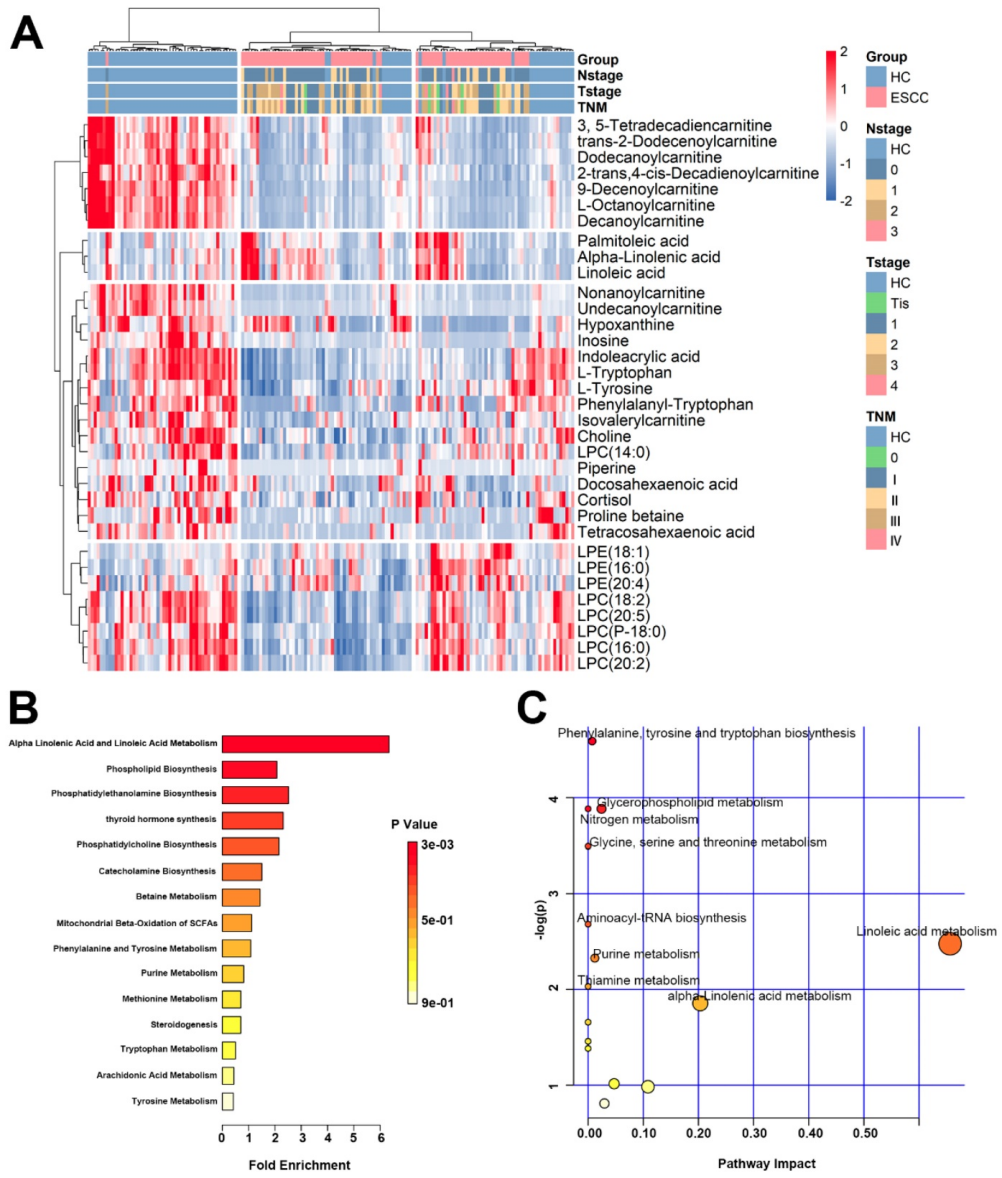
** Heat map and pathway enrichment analysis of differential metabolites.** (A) Heat map of the 35 differential metabolites in the validation set. (B) Pathway enrichment analysis related to the differential metabolites of ESCC. (C) Altered metabolic pathways in ESCC compared with HC.

A heat map was drawn from 34 differential metabolites present in HCs and the ESCC patients (Figure 3A). Among them, 6 metabolites were elevated and 28 decreased. Detailed information of 34 plasma metabolites is provided in the Supplementary materials (Table S2). Based on these differential metabolites, metabolic network enrichment analysis was performed by MetaboAnalyst to identify the dysregulated metabolic pathways (Figure 3B, C). According to the results, several metabolic pathways appear to be altered in ESCC patients compared with the normal population, with the most prominent being alpha linolenic acid and linoleic acid metabolism, phenylalanine and tyrosine metabolism, phenylalanine biosynthesis, phosphatidylethanolamine biosynthesis, glycerophospholipid metabolism, glycine serine, threonine metabolism, nitrogen metabolism, aminoacyl-tRNA biosynthesis, purine metabolism, and thiamine metabolism.

### Diagnostic performance of the biomarker panel for ESCC

**Figure 4:**
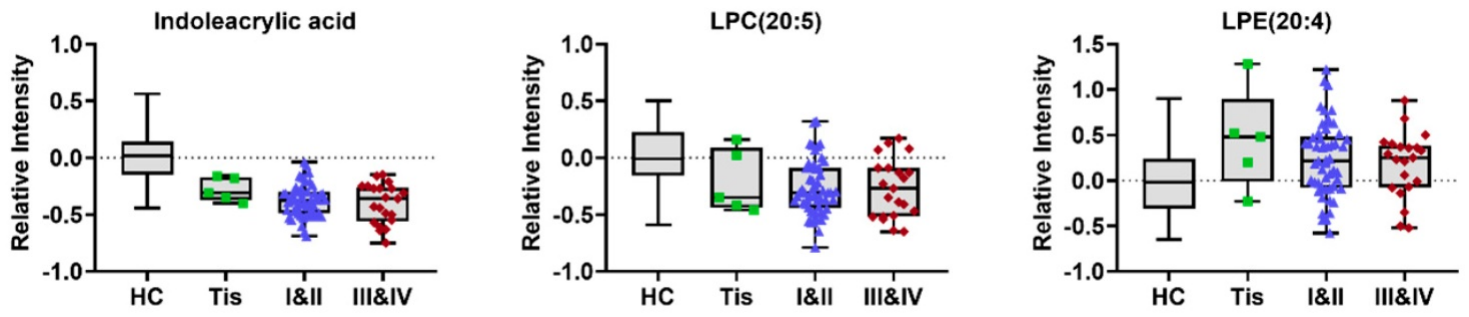
Typical variations in levels of potential metabolic biomarkers related to HC and different stages of ESCC (stage 0/Tis, I-II, III- VI).

To construct an effective diagnostic model for ESCC, we applied logistic regression analysis using the data from the training set. First, binary logistic regression analysis and an optimized algorithm of the forward stepwise method (Wald) method were applied to establish the best model using the above identified 34 metabolites. Eventually, the combination of eight metabolites was defined as the ideal biomarker panel to discriminate HC from ESCC. These eight metabolites are hypoxanthine, proline betaine, indoleacrylic acid, inosine, 9-decenoylcarnitine, tetracosahexaenoic acid, LPE (20:4) and LPC (20:5), from which one was up-regulated, and seven down-regulated in ESCC patients (Table 2). Detail information on three potential biomarkers in different stages of ESCC patients (stage 0/Tis, I-II, III-VI) is shown in (Figure 4).

**Figure 5:**
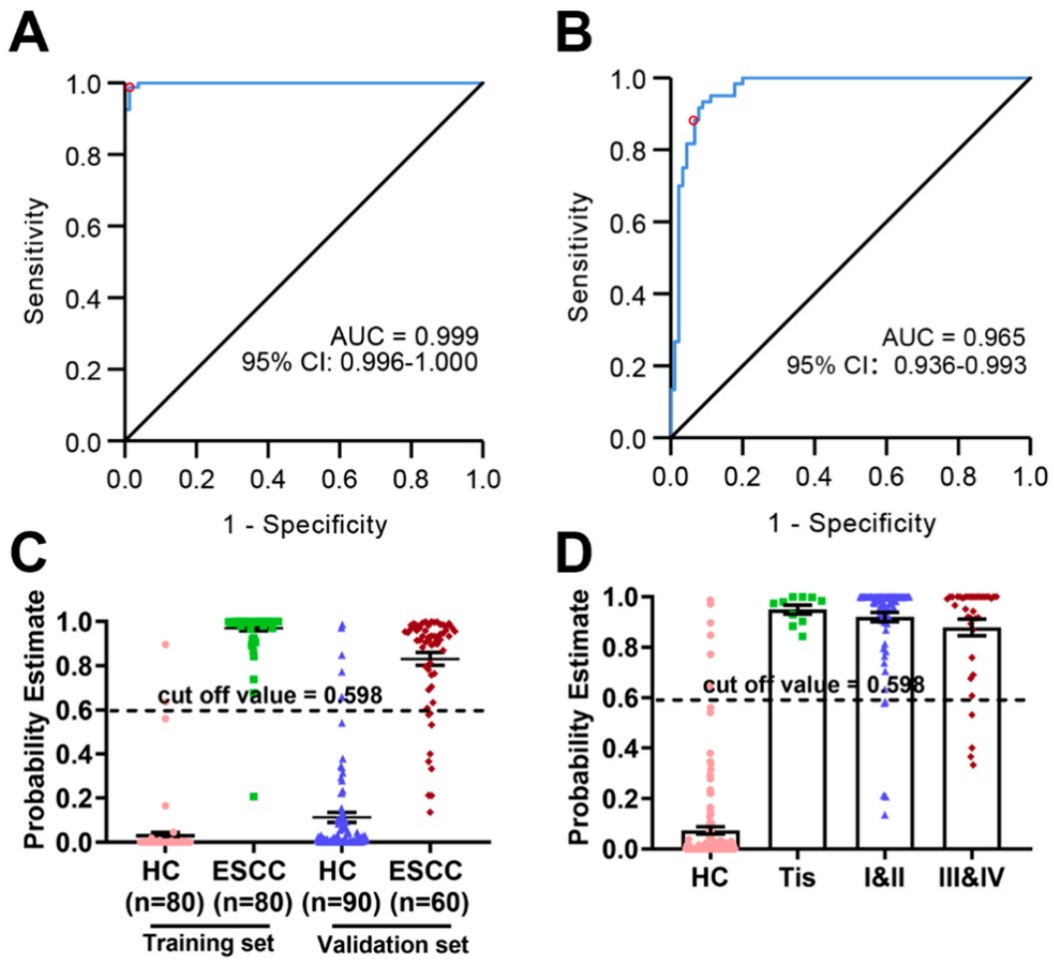
** Probability estimates based on eight metabolites from plasma.** The diagnostic outcomes in the training set (A) and validation set (B) are shown via the receiver-operating characteristic (ROC) curves for comparison between ESCC and HC. (C) Score of the ESCC biomarker signature identified in the training set and applied on the validation set. (D) Probability estimate in different stages of ESCC patients (stage 0/Tis, I-II, III-VI) relative to HC.

The diagnostic potential of these eight metabolites was evaluated in both the training set and the validation set. As shown in (Figure 5A), the AUC value of the training set was 0.991 (95% CI: 0.981-1.000, SE = 98.8%, SP = 94.9%), whereas that of the validation set was 0.965 (95% CI: 0.936-0.993, SE = 88.3%, SP = 88.9%) (Figure 5B), indicating excellent performance in classification. According to the highest prediction values of SE and SP of the ROC in the training set, the optimal cut-off value to distinguish between HC and ESCC was set to 0.598. The probability estimates in the training set (n = 160) and validation set (n = 150) provided by the metabolomics-based biomarker panels indicates significant discrimination (Figure 5C). Moreover, scatter plot analysis suggests a trend associated with the probability estimate in different stages of ESCC patients (stage 0/Tis, I-II, III-VI) relative to HC (Figure 5D).

## Discussion

In this study, we compared the metabolic profiling of HCs and ESCC patients by a non-targeted metabolomics analysis based on UPLC-QTOF/MS and obtained a series of differential metabolites. Then, we conducted an in-depth analysis of the biological function of these potential markers to identify differential metabolic pathways. Finally, we constructed an ESCC diagnostic model, which achieved high SE and SP and may be a promising clinical tool for the diagnosis of ESCC. On the basis of these results, we can easily deduce that significant changes related to purine metabolism (hypoxanthine, inosine), energy metabolism (fatty acid metabolism and amino acid metabolism), and LPCs metabolism occurr in ESCC patients.

A previous study showed that the plasma level of acylcarnitine were significantly lower in ESCC patients than in HCs 23. Another study conducted by Xu et al. 24 also showed that serum levels of acylcarnitines (octanoylcarnitine, nonanoylcarnitine, decanoylcarnitine, and undecanoylcarnitine) were significantly lower in patients with ESCC. Additionally, many cancer-related abnormalities in energy metabolism and intermediate metabolic disorders are also closely due to abnormal levels of acylcarnitine. Our results further confirmed that dysregulated plasma levels of fatty acid-related acylcarnitines may underly the decreased activity of Acetyl-CoA dehydrogenase and the disorder of long-chain fatty acid oxidation observed in ESCC patients.

Glycerophospholipid metabolism was identified as an important pathway in ESCC patients. The levels of 5 LPCs were decreased in the ESCC patients, whereas the levels of 3 LPEs were increased. Our results suggest the existence of disorders of LPC and LPE metabolism in ESCC patients. LPCs and LPEs are products or metabolites of phospatidycholines (PCs) and phosphatidyethanolamines (PEs), respectively, which are structural components of animal cell membranes. In a previous study, Kuhn, T et al. 25 revealed that lower levels of LPCs are consistently related to higher risks of breast, prostate, and colorectal cancer. In another study the lipid content of gastric cancer tissue was compared to that of adjacent non-neoplastic mucosa by imaging mass spectrometry, and Uehara, T et al. 26 demonstrated that the imbalance between LPC and PC due to overexpression of lysophosphatidylcholine acyltransferase 1 (LPCAT1) is associated with the development of gastric cancer. These changes in lipid content may influence membrane fluidity and signal transduction in cancer cells, thereby playing important roles in tumorigenesis or cancer progression. In addition, the decreased levels of LPC (14:0) in the plasma of ESCC patients was also reported by Xu et al. 24. Furthermore, LPEs can inhibit phospholipase D (PLD) and may play a crucial role in cell proliferation and migration 27. In our study, higher levels of LPE (16:0), LPE (20:4) and LPE (18:1) were found in the ESCC group. In summary, abnormalities in the plasma levels of phospholipids in ESCC patients indicated disturbed lipid and energy metabolism.

Our study also provided evidence for cell membrane permeability damage in ESCC patients. Indoleacrylic acid (IAA) is a metabolite of tryptophan, which affects the level of unsaturated fatty acids in the membrane by regulating cell lysophospholipase activity 28. For this reason, significant changes in the ratio of PE to PC and low IAA levels may indicate cellular membrane permeability damage in ESCC patients 29. Additionally, there is evidence that IAA has a role in promoting intestinal epithelial barrier function and reducing inflammation. Stimulation of IAA production can promote an anti-inflammatory response and has therapeutic benefits in inflammatory bowel disease 30. Based on this observation we hypothesize that the decrease of IAA leads to intestinal epithelial barrier dysfunction and an inflammatory response, which in turn promotes the development of ESCC. Furthermore, the observation that the decreases in IAA with the progression of ESCC was gradual suggest that IAA may be an excellent stage-related biomarker of ESCC.

In addition, the plasma levels of L-tryptophan were lower in ESCC patients. Previous studies have shown that an increase in tryptophan catabolism is associated with a decrease in T cell proliferation and a decrease in indoleamine-2,3-dioxygenase (IDO)-mediated immune response 31, 32. Although kynurenine was not detected in this study, we found low levels of IAA, a catabolic product of tryptophan through the serotonin pathway. Contrary to our results, Xu et al., reported that the metabolisms of isoleucine, arginine, methionine, tryptophan, and tyrosine was upregulated 19. These different results may be attributed to the age, gender, and cancer staging differences in the ESCC population recruited. Moreover, phenylalanyl-tryptophan, as a donor of tryptophan that is mainly metabolized in the gut, is lower in ESCC than HC. Overall, results of low levels of IAA, L-tryptophan and phenylalanyl-tryptophan may indicate that the tryptophan metabolism was altered by gut microbiota during the development of ESCC. However, the detailed mechanism should be further studied.

In the present study, abnormal metabolism of hypoxanthine and inosine (purine metabolism) was also observed. Most studies have noticed that enzymes associated with the purine biosynthetic pathway are enhanced in tumor cells because purine nucleotides are essential for tumor cell proliferation 33. Decreased serum levels of hypoxanthine and inosine have been reported in gastric cancer and other diseases 34, 35. It has been suggested that the higher propagation rate of tumor cells leads to decreased serum levels of inosine and hypoxanthine.

Elevated linoleic acid (LA) and alpha-linolenic acid (ALA) levels were also detected in ESCC patients. The pathway of LA metabolism has become a hot topic in recent research. This pathway, which contains both LA and ALA, is vital for normal physiological function, cellular function and signaling, and the immune response 36. A series of studies has shown that dysregulation of LA metabolism takes place in a variety of malignant diseases 17, 24, 37. However, the separate roles of the LA and ALA in ESCC risk remain unclear and our understanding of them incomplete. Therefore, the underlying mechanisms of LA metabolism in cancer development also requires more in-depth research.

Although many papers from many disciplines have introduced novel biomarker candidates for ESCC detection and diagnosis, most of them have not been properly verified, externally and/or internally. The ESCC patients in this study included a wide range of stages, from precancerous lesions to advanced, with a large sample size. This study revealed multiple significant disease-associated alterations in the metabolome profile with promising diagnostic power (the AUC values for the training set and validation set were 0.991 and 0.964, respectively). However, there are some limitations in this study. First, the novel biomarkers identified are based on the untargeted profiling analyses, and the generated hypotheses therefore require verification by absolute quantification. Moreover, it should be noted that this study was performed only in one center and the diagnostic power of the biomarkers needs to be verified by a large-scale external cohort. Third, to obtain a wide metabolome coverage, a dual analytical platform, such as gas chromatography-mass spectrometry in combination with LC/MS, is recommended for future research.

## Conclusion

In this UPLC-QTOF/MS-based metabolomics study, different metabolic profiles were discovered in ESCC and HC plasma, which might form the basis of a non-invasive approach for the diagnosis of the disease, based on the identified differential biomarkers. There were 34 metabolites with altered plasma levels in ESCC patients, and among these, eight metabolites namely hypoxanthine, proline betaine, indoleacrylic acid, inosine, 9-decenoylcarnitine, tetracosahexaenoic acid, LPE (20:4), and LPC (20:5), were significantly dysregulated. The combination of these eight biomarkers exhibited a high diagnostic value. Accordingly, energy metabolism (amino acid metabolism and fatty acid metabolism), purine metabolism (hypoxanthine, inosine), and LPCs metabolism appear to be highly associated with ESCC.

## Supplementary Material

Supplementary figures and tables.

## Figures and Tables

**Table 1:** Clinical characteristics of the subjects in the training set and validation sets.

	Training set(n=160)			validation set(n=150)		
	case	control	*p*	case	control	*p*
**number**	80	80		60	90	
**Anthropometric characteristics**						
**age (years)**	59.40±8.08	51.45±5.55	<0.0001	60.43±8.14	51.58±8.03	<0.0001
**gender (M/F)**	53/27	45/35	0.194	41/19	52/38	0.192
**BMI (kg/m^2^)**	22.74±2.93	24.71±3.40	<0.0001	21.89±3.35	25.14±3.38	<0.0001
**Blood Parameters**						
**WBC (10^9/L)**	6.25±1.83	6.24±1.63	0.9632	6.15±1.99	6.39±1.44	0.391
**Hb (g/L)**	133.52±13.25	145.27±14.88	<0.0001	130.44±13.44	148.31±14.01	<0.0001
**PLT (10^9/L)**	205.16±56.58	233.55±51.52	0.0012	206.02±55.73	239.64±53.35	0.0003
**TNM stages**						
**High-grade dysplasia**	5			5		
**Ⅰ**	23			16		
**ⅠⅠ**	31			24		
**ⅠⅠⅠ**	19			11		
**IV**	2			4		

Unpaired t tests for continuous measures and χ2 tests for categorical variables. Abbreviations: BMI, body mass index; WBC, white blood cell; Hb, hemoglobin; PLT, platelet count.

**Table 2:** Potential plasma biomarkers associated with ESCC.

				AUC		
metabolites	VIP	adj *p*	FC	training set	validation set	biological pathway
Hypoxanthine	4.41	4.05E-08	0.46	0.794	0.780	Purine metabolism
Proline betaine	2.95	4.10E-07	0.29	0.763	0.670	Amino acids metabolism
Indoleacrylic acid	6.20	1.17E-21	0.63	0.913	0.677	Amino acids metabolism
Inosine	2.51	1.33E-08	0.18	0.895	0.619	Purine metabolism
9-Decenoylcarnitine	6.60	7.34E-19	0.34	0.944	0.873	Fatty acids metabolism
Tetracosahexaenoic acid	1.70	1.77E-10	0.17	0.904	0.781	Fatty acids metabolism
LPE(20:4)	3.32	1.59E-03	1.25	0.668	0.666	phospholipid metabolism
LPC(20:5)	12.60	2.41E-07	0.75	0.769	0.770	phospholipid metabolism

Abbreviations: ESCC, esophageal squamous cell carcinoma; AUC, area under the curve; VIP, variable importance in the projection; adj *p*, adjusted *p* value; FC, fold change; LPC, lysophosphatidylcholine; LPE, lysophosphatidylethanolamine.

## Abbreviations

EC: Esophageal cancer
ESCC: Esophageal squamous cell carcinoma
HC: Healthy control
UPLC-QTOF/MS: Ultra-performance liquid chromatography-tandem quadruple time-of-flight mass spectrometry
AUC: Area under curve
ROC: Receiver operating characteristic curve
CI: Confidence interval
SE: Sensitivity
SP: Specificity
NMR: Nuclear magnetic resonance
LC-MS: Liquid chromatography-mass spectrometry
AJCC: American Joint Committee on Cancer
QC: Quality control
ESI: Electrospray ionization
CDS: Calibrant delivery system
IDA: information dependent acquisition
DP: Declustering potential
CE: Collision energy
CES: Collision energy spread
DBS: Dynamic background subtraction
MVA: Multivariate analysis
PCA: Principal component analysis
OPLS-DA: Orthogonal partial least squares discriminant analysis
VIP: Variable importance in the projection
FC: Fold changes
WBC: White blood cell
Hb: Hemoglobin
PLT: Platelet count
SD: Standard deviation
RPT: Response permutation testing
LPC: Lysophosphatidylcholine
LPE: Lysophosphatidylethanolamine
LPCAT1: Lysophosphatidylcholine acyltransferase 1
PLD: Phospholipase D
IAA: Indoleacrylic acid
IDO: Indoleamine-2,3-dioxygenase
LA: Linoleic acid
ALA: alpha-linolenic acid
