# Supplementary material for: Untargeted Metabolomics Analysis of Esophageal Squamous Cell Carcinoma Discovers Dysregulated Metabolic Pathways and Potential Diagnostic Biomarkers
Source: J Cancer. 2020 Apr 6;11(13):3944–54. doi: 10.7150/jca.41733 (PMC7171502; doi:10.7150/jca.41733)
Supplement: Supplementary file 1 — Supplementary figures and tables. [file jcav11p3944s1.pdf]

Supplement Tables

Table S1. The confirmation of the chemical structures of 11 serum metabolites using standard references.

Identify Name	Polarity	Measured m/z	Measured RT (min)	Accurate m/z	Standard RT (min)	m/z error (ppm)	RT error (min)
Hypoxanthine	POS	137.0447	1.56	137.0466	1.53	13.86	0.03
Indoleacrylic acid	POS	188.0701	1.97	188.0709	2.01	-4.25	-0.04
Isovalerylcarnitine	POS	246.1703	2.18	246.1688	2.25	6.09	-0.07
Inosine	POS	269.0885	1.59	269.0877	1.63	2.97	-0.04
Piperine	POS	286.1429	4.52	286.1425	4.48	1.40	0.04
Cortisol	POS	363.2164	3.14	363.2158	3.17	1.65	-0.03
L-Tyrosine	POS	182.0806	1.47	182.0805	1.51	0.55	-0.04
L-Tryptophan	POS	205.0969	1.97	205.0976	1.98	-3.41	-0.01
Linoleic acid	POS	281.2476	9.77	281.2394	9.63	29.16	0.14
Docosahexaenoic acid	POS	329.2402	9.30	329.2368	9.19	10.33	0.11
alpha-Linolenic acid	POS	279.1617	7.78	279.1586	7.07	11.1	0.71

Retention time (min, RT); Measured mass to charge ratio (m/z). m/z error was calculated using (Measured m/z – Standard m/z) *106/ Standard m/z, and RT error was calculated using (Measured RT – Standard RT).

Table S2. List of metabolites in plasma samples from ESCC patients that were significantly different than in HC samples.

NO.	name	m/z (Da)	Rt (min)	VIP	adj.p	FC
1	Choline	104.1	0.7	1.11	8.43E-07	0.82
2	Hypoxanthine	137.0	1.6	4.41	4.05E-08	0.46
3	Proline betaine	144.1	0.8	2.95	4.10E-07	0.29
4	L-Tyrosine	182.1	1.5	1.62	3.78E-04	0.80
5	Indoleacrylic acid	188.1	2.0	6.20	1.17E-21	0.63
6	L-Tryptophan	205.1	2.0	5.10	3.12E-21	0.61
7	Isovalerylcarnitine	246.2	2.1	1.02	1.68E-09	0.46
8	Palmitoleic acid	255.2	9.3	1.27	3.54E-02	1.39
9	Inosine	269.1	1.6	2.51	1.33E-08	0.18
10	Alpha-Linolenic acid	279.2	8.8	3.40	6.95E-03	1.42
11	Linoleic acid	281.2	9.8	4.08	3.68E-02	1.27
12	Piperine	286.1	4.5	3.70	3.11E-03	0.10
13	L-Octanoylcarnitine	288.2	2.9	4.50	2.65E-15	0.35
14	Nonanoylcarnitine	302.2	3.1	1.45	1.51E-14	0.15
15	2-trans,4-cis-Decadienoylcarnitine	312.2	3.0	1.78	3.75E-13	0.41
16	9-Decenoylcarnitine	314.2	3.2	6.60	7.34E-19	0.34
17	Decanoylcarnitine	316.2	3.4	5.92	7.88E-17	0.33
18	Docosahexaenoic acid	329.2	9.3	1.89	1.64E-03	0.67
19	Undecanoylcarnitine	330.3	3.6	1.37	7.76E-09	0.12
20	trans-2-Dodecenoylcarnitine	342.3	3.6	2.30	1.00E-09	0.48
21	Dodecanoylcarnitine	344.3	3.9	2.39	2.53E-10	0.45
22	Phenylalanyl-Tryptophan	352.2	2.4	1.57	4.41E-05	0.51
23	Tetracosahexaenoic acid	357.3	4.6	1.70	1.77E-10	0.17
24	Cortisol	363.2	3.1	1.51	4.69E-04	0.66
25	3, 5-Tetradecadiencarnitine	368.3	3.8	2.43	2.52E-05	0.59
26	LPE(16:0)	454.3	5.8	2.09	5.24E-04	1.33
27	LPC(14:0)	468.3	4.7	3.73	1.38E-04	0.62
28	LPE(18:1)	480.3	6.2	2.98	2.16E-02	1.42
29	LPC(16:0)	496.3	6.0	22.84	1.34E-03	0.82
30	LPE(20:4)	502.3	5.4	3.32	1.59E-03	1.25
31	LPC(P-18:0)	508.4	6.9	1.66	1.25E-02	0.77
32	LPC(18:2)	520.3	5.5	28.57	9.01E-08	0.70
33	LPC(20:5)	542.3	5.4	12.60	2.41E-07	0.75
34	LPC(20:2)	548.4	6.8	1.79	1.14E-03	0.72

Abbreviations: ESCC, esophageal squamous cell carcinoma; HC, healthy control; Rt, retention time; VIP, variable importance in the projection; adj p, adjusted p value; FC, fold change; LPC, lysophosphatidylcholine; LPE, lysophosphatidylethanolamine.

Supplement Figures

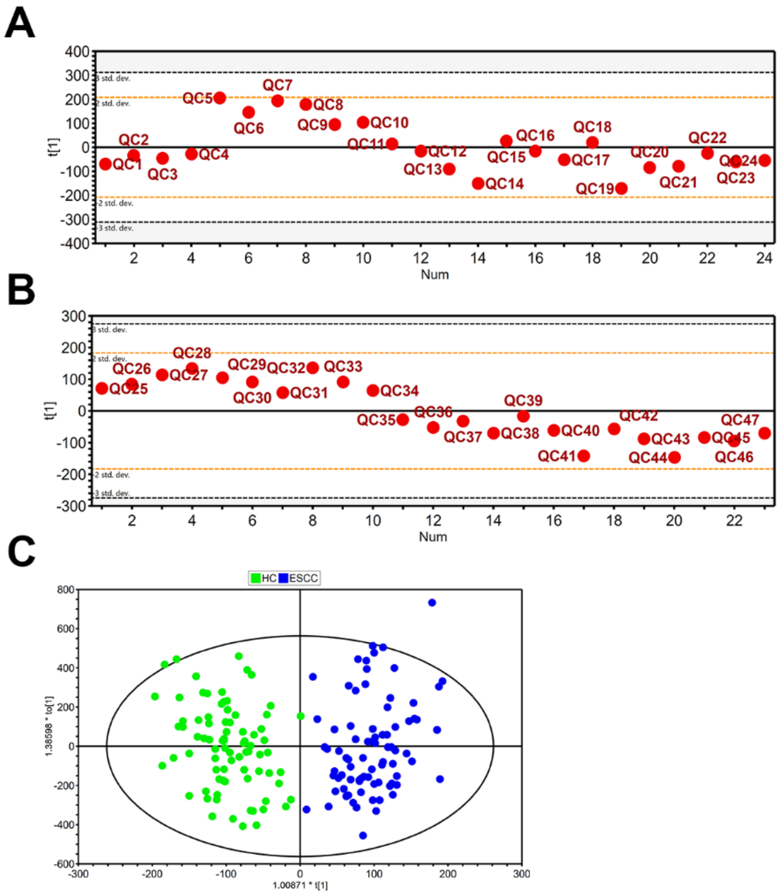

Figure S1. Stability of the analytical methods. PCA score plots of QC samples in both the training set (A) and validation set (B). (C) OPLS-DA score plot of validation set.

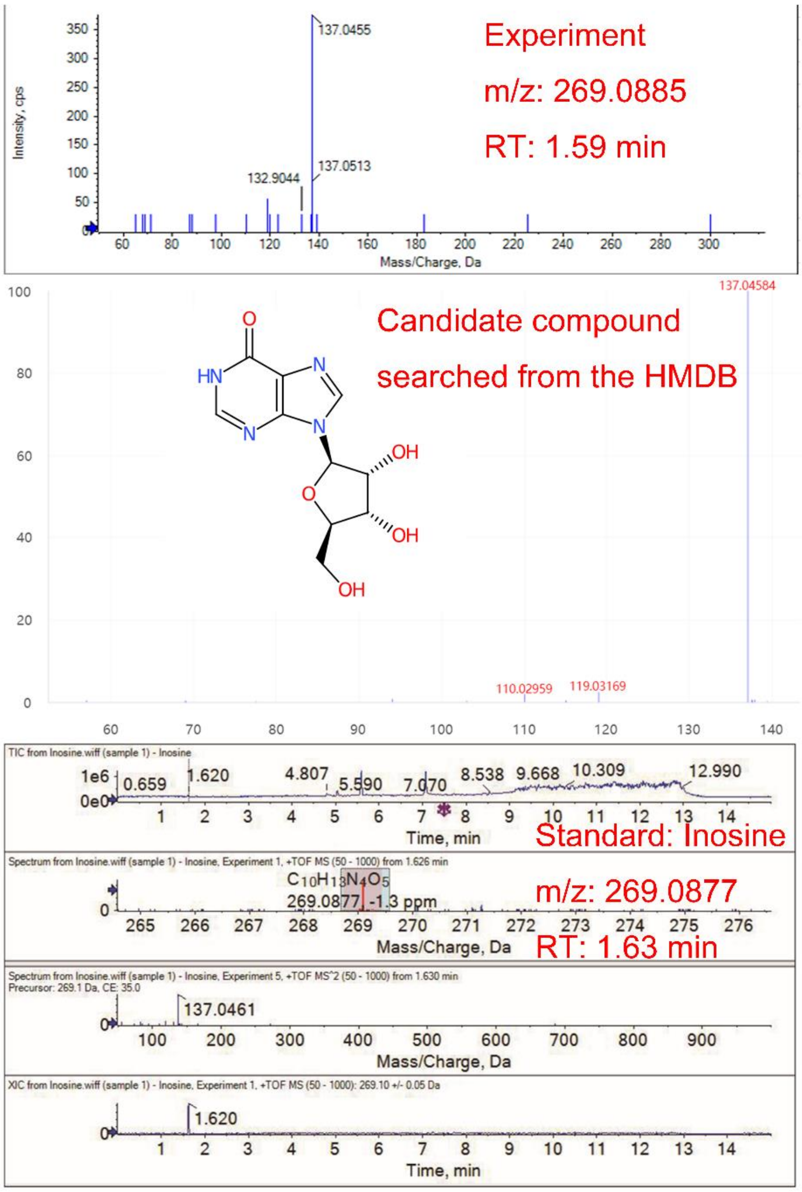

Figure S2. Metabolite identification. Take the identification of inosine as an example to explain the identification process.
